# Prognostic and diagnostic value of circRNA expression in prostate cancer: A systematic review and meta-analysis

**DOI:** 10.3389/fonc.2022.945143

**Published:** 2022-11-07

**Authors:** Jingling Xie, Hui Jiang, Yuanqing Zhao, Xin rui Jin, Baolin Li, Zixin Zhu, Limei Zhang, Jinbo Liu

**Affiliations:** Department of Laboratory Medicine, The Affiliated Hospital of Southwest Medical University, Luzhou, Sichuan, China

**Keywords:** circular RNAs, prostate cancer, diagnosis, prognosis, clinicopathological features

## Abstract

**Background:**

Circular RNAs (circRNAs) are receiving increasing attention as novel biomarkers. Our goal was to investigate the diagnostic, clinicopathological, and prognostic utility of circRNAs in prostate cancer (PCa).

**Methods:**

Relevant literature was searched in PubMed, Web of Science, and EMBASE. Sensitivity, specificity, diagnostic odds ratio (DOR), negative likelihood ratio (NLR), positive likelihood ratio (PLR), and the area under the curve (AUC) were calculated to evaluate the diagnostic accuracy of circRNA expression. circRNAs’ clinical, pathological, and prognostic value was examined using pooled odds ratios (ORs) and hazard ratios (HRs).

**Results:**

This meta-analysis included 23 studies, with 5 for diagnosis, 16 for clinicopathological parameters, and 10 for prognosis. For diagnostic value, the pooled sensitivity, pooled specificity, PLR, NLR, DOR, and AUC were 0.82, 0.62, 2.17, 0.29, 7.37, and 0.81, respectively. Upregulation of carcinogenic circRNAs was associated with poor clinical parameters (Gleason score: OR = 0.222, 95% CI: 0.145–0.340; T classification: OR = 0.274, 95% CI: 0.175–0.430; lymph node metastasis: OR = 0.353, 95% CI: 0.175–0.716; tumor size: OR = 0.226, 95% CI: 0.099–0.518) and could predict poor survival outcomes (HR = 2.408, 95% CI: 1.559–3.720, p < 0.001). Conversely, downregulation of tumor-suppressor circRNAs was also associated with poor clinical parameters (Gleason score: OR = 1.689, 95% CI: 1.144–2.493; T classification: OR = 2.586, 95% CI: 1.779–3.762) and worse prognosis (HR = 1.739, 95% CI: 1.147–2.576, p = 0.006).

**Conclusion:**

Our results showed that circRNAs might be useful biomarkers for the diagnosis and prognosis of PCa.

**Systematic review registration:**

https://www.crd.york.ac.uk/prospero/, identifier CRD42021284785.

## Introduction

Prostate cancer (PCa) is one of the most common malignancies in men worldwide, accounting for approximately 27% of all cancer cases with a high mortality rate (1). According to the global cancer statistics, in 2018, there were about 1,276,106 new cases and 358,989 death cases every year (2). Although the morbidity of PCa is lower in China than in other countries, the annual incidence has been on the rise (3). Early-stage PCa can be cured by surgery and chemotherapy, with 5-year survival rates exceeding 90%. The inhibition of gonadal testosterone production with androgen deprivation therapy has been the cornerstone of PCa treatment (4). In addition, multiple drugs, including abiraterone, enzalutamide, apalutamide, darolutamide, and docetaxel, have been approved for advanced PCa (4, 5). However, most advanced PCa can turn into castration-resistant PCa after long-term castration treatments, with 5-year survival rates, were below 30% (5, 6). Therefore, biomarkers are needed urgently for early diagnosis and for assessing the prognostic status of patients with PCa.

In recent years, prostate-specific antigen (PSA) has been used for the early determination and staging of PCa. However, its accuracy in predicting prognosis and biochemical recurrence is still questionable; thus, PSA is not recommended by experts (7, 8). Therefore, there is an urgent need to explore new biomarkers with high sensitivity, specificity, and reproducibility in the diagnosis, prognosis, and treatment of PCa.

Circular RNAs (circRNAs) are endogenous non-coding RNAs that range from a few hundred to thousands of nucleotides (9). circRNAs were once thought to be splicing faults; however, their structure was discovered because of the rapid growth of whole-genome sequencing (10). The circRNAs’ 3′ and 5′ ends covalently form a loop, without the 5′ cap and 3′ poly(A) tails, making them structurally conserved and stable (11, 12). circRNAs are implicated in a variety of biological activities (13), including microRNA or protein sequestration, transcription regulation, splicing interference, and polypeptide translation (14). In addition, they play a role in physiological illnesses such as cancer cell proliferation, differentiation, apoptosis, and metastasis (15). Meanwhile, circRNAs are increasingly linked to the incidence and progression of PCa. Wang et al. (16) discovered that high levels of hsa_circ 0088233 increased the malignant phenotypes of PCa by sequestering miR-515-5p and inducing FKBP1A expression. Xia et al. explored the diagnostic value of circ_0057558 and circ_0062019 in PCa (17). Moreover, the prognostic role of circ_PSMC3 in PCa has also been explored in other studies (18). However, detailed discussions on the role of circRNAs in diagnostic and prognostic value are still lacking. In the present meta-analysis, we included papers on the involvement of circRNAs in PCa and investigated their potential diagnostic, clinicopathological, and prognostic relevance.

## Materials and methods

### Registration

The protocol was registered on the Prospective Register of Systematic Reviews (PROSPERO) with registration number CRD42021284785.

### Data search strategy

Our literature search was guided by the recently published PRISMA statement (19, 20).

We searched all relevant articles through PubMed, Embase, and Web of Science online databases that were published before 8 October 2021.

To avoid omission, two independent researchers completed the retrieval process by combing Medical Subject Heading (MeSH) terms and text words. For literature retrieval, the following MeSH terms and text words were used: (1) “Prostatic Neoplasms”, “Prostate Cancer”, or “PCa”; (2) “RNA”, “Circular”, or “circRNAs”. The detailed search strategy is shown in **Supplementary Material**. The language was limited to English. In addition, the references of the identified studies were also searched for relevant documents. Other details are provided in the **Supplementary Material**. The authors of the included articles were contacted when deemed necessary.

### Inclusion and exclusion criteria

Two independent investigators assessed the appropriate studies and extracted the imperative data, and disagreements were resolved by discussing with the third researcher. Studies were included if they assessed the accuracy of circRNA for differentiation between PCa and non-PCa patients. To be eligible, studies needed a clearly defined standard of reference. We defined PCa according to the guidelines of the European Association of Urology, the International Society of Geriatric Oncology (21), and the National Comprehensive Cancer Network (22).

The inclusion criteria were as follows: (1) patients with a pathological diagnosis of PCa; (2) expression level of circRNAs could be divided into high and low expression; (3) studies that included data to estimate the diagnostic, prognostic, and clinicopathologic features; and (4) cohort or case-control research.

The exclusion criteria are as follows: (1) duplicate studies; (2) reviews, meta-analyses, letters, conference abstracts, or case reports; (3) articles without complete information; (4) for diagnostic meta, those without clear tests and control group size, and those without true positive (TP), true negative (TN), false positive (FP), and false negative (FN) or sensitivity (SEN), specificity (SPC), and receiver operating characteristics (ROC); and (5) for the prognostic meta-analysis, those without clear information on the number of trial and control groups, survival information, and Kaplan-Meier (KM) plots.

### Data extraction

Two researchers extracted the data independently. The extracted information was as follows: (1) first author, publication year, type of cancer, circRNAs, numbers of patients, detection methods, and outcomes; (2) follow-up time and outcomes; (3) sensitivity, specificity, and the areas under the curve (AUCs) of circRNAs for diagnosis; and (4) clinicopathological features including age, smoking, drinking, TNM stage, T classification, lymph node metastasis, distant metastasis, and Gleason score. If the HRs with 95% CIs for outcomes were not shown directly in the article, then the survival data were extracted from KM plots by Engauge Digitizer 4.1 software. The HRs and 95% CIs were calculated using the Excel program file provided by Tierney et al. (23). If the parameters of TP, TN, FP, and FN were not offered, then we assessed them according to sample size, SPC, SEN, and AUC.

### Quality assessment

Quality Assessment of Diagnostic Accuracy Studies-2 (QUADAS-2) and Newcastle Ottawa Scale (NOS) were used by two independent investigators to assess the quality of the studies for diagnosis and prognosis, respectively. The four domains of QUADAS-2 were as follows: patient selection, index test, reference standard, and flow and timing. Bias risk was graded as high (H), low (L), or unclear (U). The total scores of NOS ranged from 0 to 8, and the studies that were higher than 6 in NOS were considered high-quality studies.

### Statistical analysis

Review Manager 5.3 and Stata 15.0 were used for statistical analysis. I^2^ statistics were used to perform the heterogeneity test. We determined that there was considerable heterogeneity among the included studies if the I^2^ value > 50% and the p-value < 0.05. Random-effects model was used to examine the pooled results. If there was no significant heterogeneity in the included studies, then a fixed-effects model was used. A p-value < 0.05 was used to determine statistical significance.

In the diagnostic meta-analysis, the number of TP, FP, FN, and TN was combined to calculate pooled results of sensitivity, specificity, diagnostic odds ratio (DOR), negative likelihood ratio (NLR), and positive likelihood ratio (PLR). The area under the summary ROC (SROC) was calculated to determine the diagnostic accuracy of circRNA expression. Deeks’ funnel plot asymmetry test was used to investigate potential publishing bias. If the p-value > 0.1, then we considered that there was no publishing bias. Pooled ORs and 95% CIs were used to explore the association between circRNA expression and clinicopathological features. For the prognostic meta-analysis, HRs and 95% CIs were used to assess the prognostic value of circRNAs. Egger’s tests were used to determine the possibility of publication bias. To determine the stability of the pooled HR, a sensitivity analysis was done. If the p-value > 0.1 for Egger’s tests, then we considered that there was no publication bias.

## Results

### Search results

**Figure 1:**
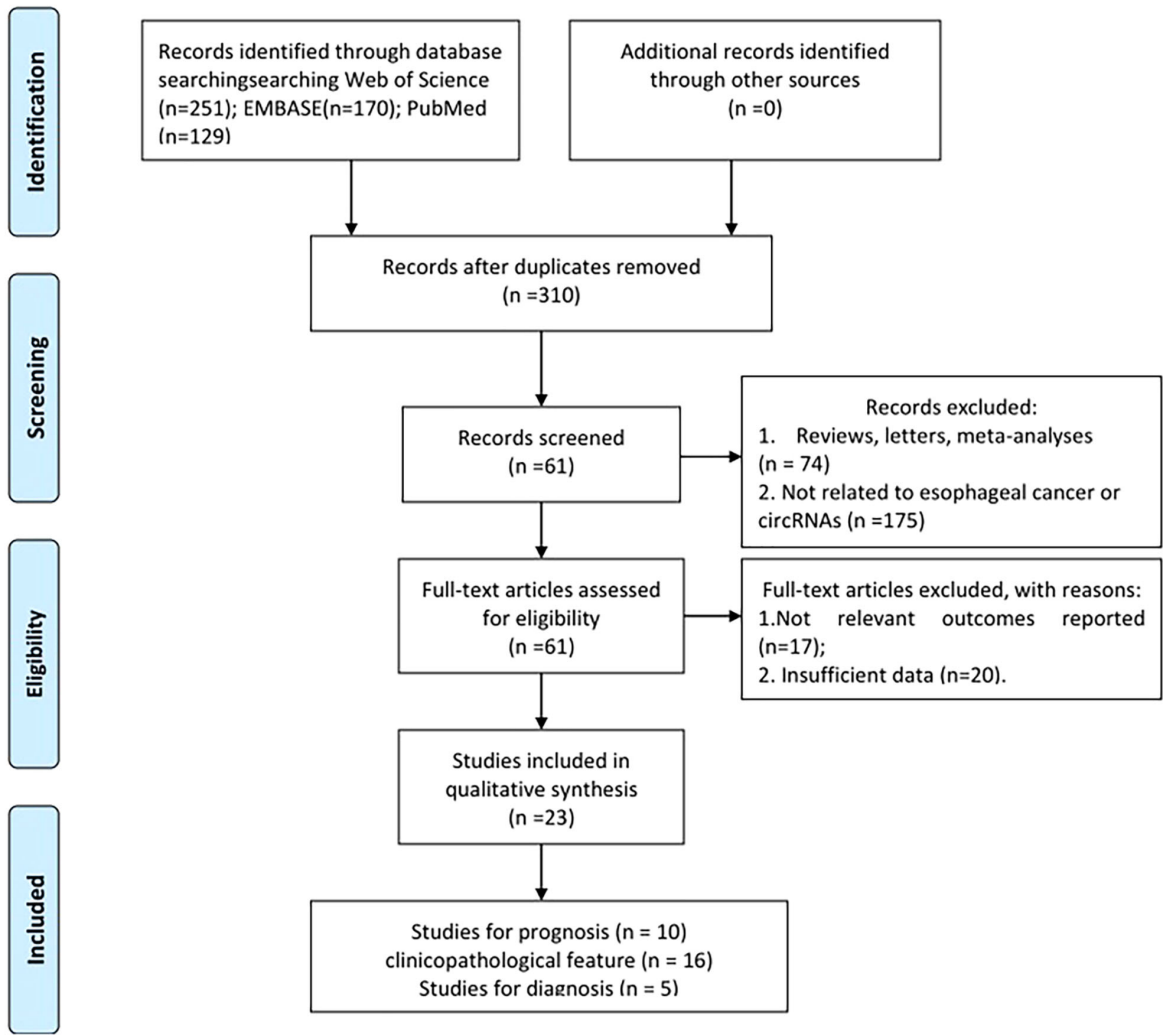
Flowchart of trial selection.

The flow diagram for study selection is given in **Figure 1**. A total of 550 relevant studies were found from PubMed (129 records), Embase (170 records), and Web of Science (251 records) from initial screening. After eliminating duplicate items, 310 articles were obtained. Furthermore, 249 articles were filtered out for inappropriate types (175 irrelevant articles and 74 reviews, letters, or meta-analyses). After the review of full-text articles, 37 articles were excluded for the following reasons: 17 did not include relevant outcomes and 20 did not report complete data. Finally, 23 studies ranging from 2018 to 2021 were screened for meta-analysis, including 5 for diagnosis, 16 for clinicopathological features, and 10 for prognosis (1, 14, 16–18, 24–40).

### Study characteristics and quality assessment

**Figure 2:**
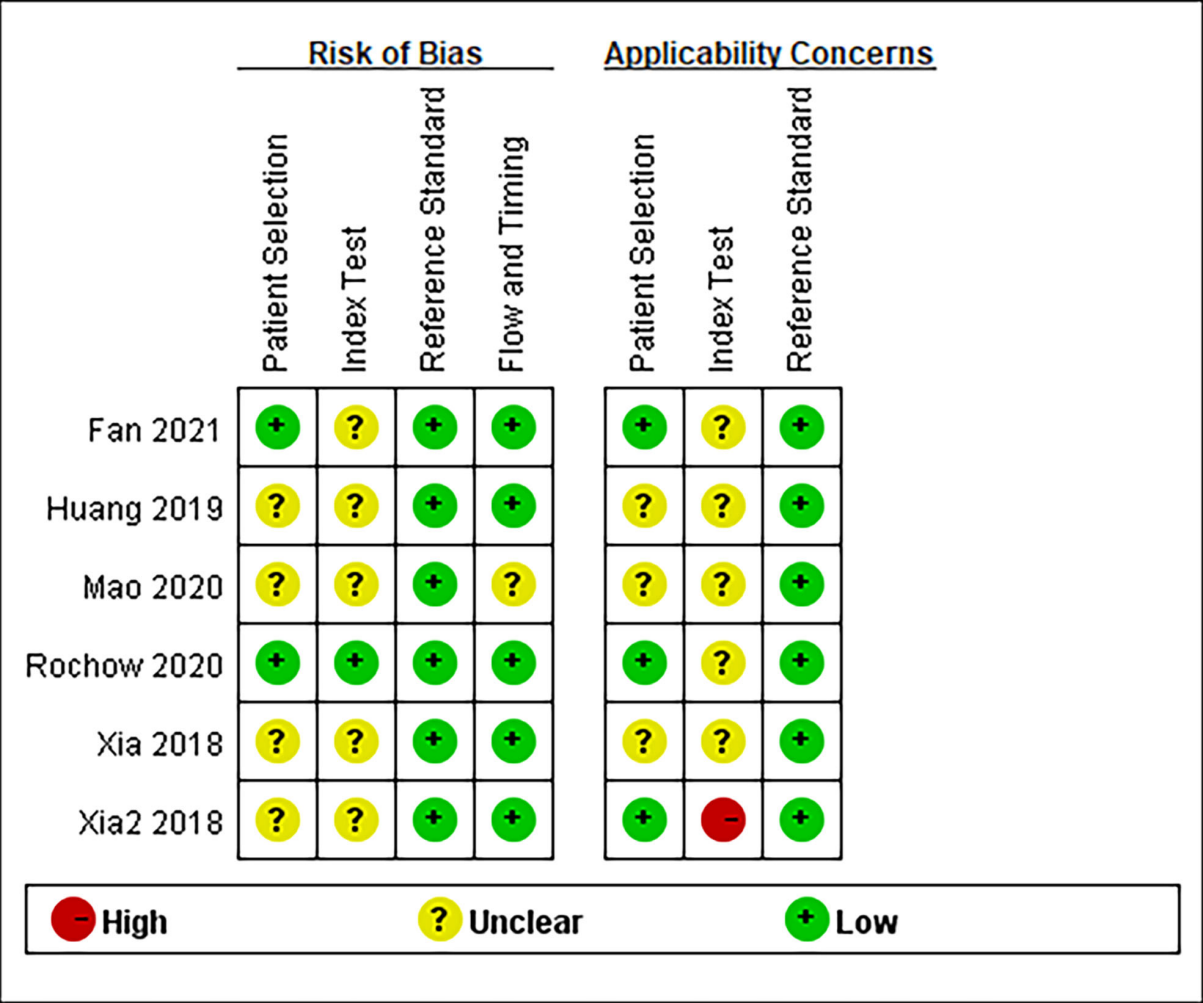
Quality assessment of eligible studies for diagnostic meta-analysis.

The essential characteristics of the included studies are shown in **Tables 1**–**4**. A total of 23 circRNAs were included and published between 2018 and 2021. Quantitative real-time reverse transcription PCR (qRT-PCR) was used to calibrate the expression of circRNAs. The majority of the included studies were from China. Among them, some elements employed in the diagnostic analysis, such as sensitivity, specificity, and AUC, are recorded in **Table 1**. The range of sample size was between 112 and 324. The quality of the contained literature was evaluated in terms of bias risk and applicability concerns. The results indicated that the quality of our included research was good (**Figure 2**).

**Table 1:** Basic features of studies for diagnosis analysis.

					Sample size				Diagnosis power		
Study	Year	Country	circRNA	Cancer type	Case	Control	Sample type	Methods	Sen	Spe	AUC
Xia1 et al.	2018	China	circ_0057558	PCa	95	78	Tissue	qRT-PCR	74.10	63.40	0.729
Xia2 et al.	2018	China	circ_0062019	PCa	95	78	Tissue	qRT-PCR	80.00	74.60	0828
Huang et al.	2019	China	circ-ITCH	PC	162	162	Tissue	qRT-PCR	88.30	61.70	0.8112
Fan et al.	2021	China	circSOBP	PCa	56	56	Tissue	qRT-PCR	83.90	40.00	0.763
Mao et al.	2020	China	hsa_circ_0003768 (circPDHX)	PCa	75	75	Tissue	qRT-PCR	80.00	58.70	0.64
Rochow et al.	2020	Germany	circATXN10	PCa	115	79	Tissue	qRT-PCR	77.00	72.00	0.801

AUC, area under the ROC curve; qRT-PCR, quantitative real-time polymerase chain reaction; Sen, sensitivity; Spe, specificity; PCa, prostate cancer; circRNAs, circular RNAs.

**Table 2:** Clinical parameters of circRNAs in PCa.

	Tumor promoter			Tumor Suppressor		
	OR	95% CI	P	OR	95% CI	P
Age (older/younger)	0.883	0.629–1.238	0.470	1.256	0.882–1.788	0.206
Gleason score	0.222	0.145–0.340	0.000	1.689	1.144–2.493	0.008
TNM stage (III + IV/I + II)	1.228	0.637–2.365	0.540	1.088	0.243–4.861	0.912
T classification (T3 + T4/T1 + T2)	0.274	0.175–0.430	0.000	2.586	1.779–3.762	0.000
Lymph node metastasis (Y/N)	0.353	0.175–0.716	0.004	1.502	0.988–2.284	0.057
Distant metastasis (Y/N)	0.823	0.181–3.741	0.801	1.300	0.330–5.115	0.707
PSA	0.632	0.213–1.881	0.410	1.054	0.729–1.524	0.778
Smoking	1.364	0.457–4.071	0.578	2.250	0.724–6.989	0.161
Tumor size	0.226	0.099–0518	0.000	–	–	–

CI, confidence interval; N, no; Y, yes; circRNAs, circular RNAs; OR, odds ratio. The results are in bold if p < 0.05.

**Table 3:** Basic features of studies for prognosis analysis.

Author	Year	Country	circRNA	Cancer type	circRNA expression		Methods	Sample type	Regulation	Follow-up (month)	HR	CI
					High	Low						
Dong et al.	2020	China	circPSMC3	PCa	55	55	qRT-PCR	Tissue	Downregulated	62	2.48	1.10–5.63
Liu et al.	2021	China	CircFOXM1	PCa	26	26	qRT-PCR	Tissue	Upregulated	62	3.01	1.36–6.66
Hu et al.	2020	China	Circ‐MTO1	PCa	298	298	qRT-PCR	Tissue	Downregulated	48	1.56	0.96–2.53
Liu et al.	2020	China	circHIPK3	PCa	28	28	qRT-PCR	Tissue	Upregulated	62	1.52	0.54–4.30
Huang et al.	2019	China	circABCC4	PCa	24	23	qRT-PCR	Tissue	Upregulated	62	3.34	0.94–12.57
Tao et al.	2019	China	circABCC4	PCa	21	21	qRT-PCR	Tissue	Upregulated	100	4.94	0.84–29.12
Wang et al.	2019	China	CircITCH	PCa	26	26	qRT-PCR	Tissue	Downregulated	80	1.58	0.49–5.15
Gao et al.	2020	China	hsa_circ_0000735	PCa	25	25	qRT-PCR	Tissue	Upregulated	62	3.03	1.11–8.22
Mao et al.	2020	China	hsa_circ_0003768 (circPDHX)	PCa	54	21	qRT-PCR	Tissue	Upregulated	105	1.27	0.19–8.34
Li et al.	2020	China	circ-0016068	PCa	42	42	qRT-PCR	Tissue	Upregulated	62	1.22	0.33–4.49

PCa, prostate cancer; qRT-PCR, quantitative real-time polymerase chain reaction; CI, confidence interval; HR, pooled hazard ratio.

**Table 4:** Quality assessment of eligible studies for clinical parameter analysis and prognosis analysis according to the Newcastle-Ottawa Scale.

Study	Selection			Comparability				Outcome		Total
	Adequacy of case definition	Number of case	Representativeness of the cases	Ascertainment of relevant cancers		Ascertainment of detection method	circRNA expression	Assessment of outcome	Adequate follow-up	
Wang et al.	1	1	1		1	1	1	0	0	6
Liu et al.	1	1	1		1	1	1	1	1	8
Huang et al.	1	1	1		1	1	1	1	0	7
Tao et al.	1	1	1		1	1	1	1	0	7
Wang et al.	1	1	1		1	1	1	1	1	8
Li et al.	1	1	1		1	1	1	1	1	8
Shi et al.	1	0	1		1	1	1	1	0	7
Huang et al.	1	1	1		1	1	1	1	1	8
Chao et al.	1	1	1		1	1	1	0	0	6
Song et al.	1	1	1		1	1	1	0	0	6
Li et al.	1	1	1		1	1	1	1	0	7
Liu et al.	1	1	1		1	1	1	1	0	7
Cai et al.	1	1	1		1	1	1	1	0	7
Wang et al.	1	1	1		1	1	1	0	0	6
Chen et al.	1	1	1		1	1	1	1	1	8
Huang et al.	1	1	1		1	1	1	1	0	7
Dong et al.	1	1	1		1	1	1	1	1	8
Hu et al.	1	1	1		1	1	1	1	1	8
Liu et al.	1	1	1		1	1	1	1	0	7
Gao et al.	1	1	1		1	1	1	1	1	8

The relationship between clinicopathological characteristics and circRNAs is given in **Table 2**. As indicated in **Table 3**, a total of 10 circRNAs were used in 10 investigations, providing some basic information about the prognostic analysis. The patients’ follow-up time ranged from 48 to 105 months, and the number of samples collected ranged from 42 to 596. The NOS scores indicated the excellent quality of studies that were included for clinical parameter analysis and prognostic analysis on each of the eight dimensions (**Table 4**).

### Diagnosis analysis

**Figure 3:**
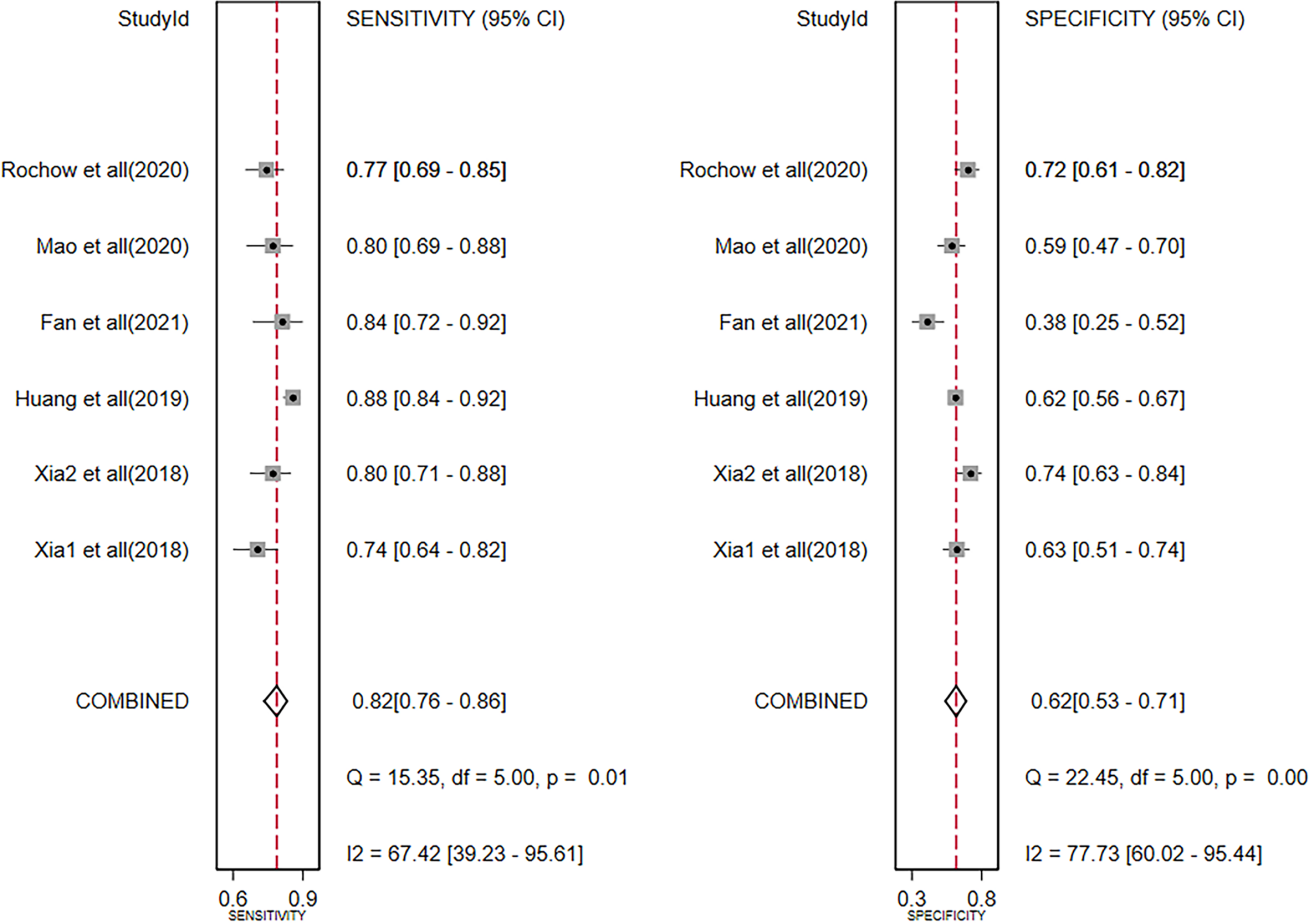
Forest plots of summary sensitivity and specificity to illustrate the diagnostic value of circRNAs for PCa. circRNAs, circular RNAs; PCa, prostate cancer.

**Figure 4:**
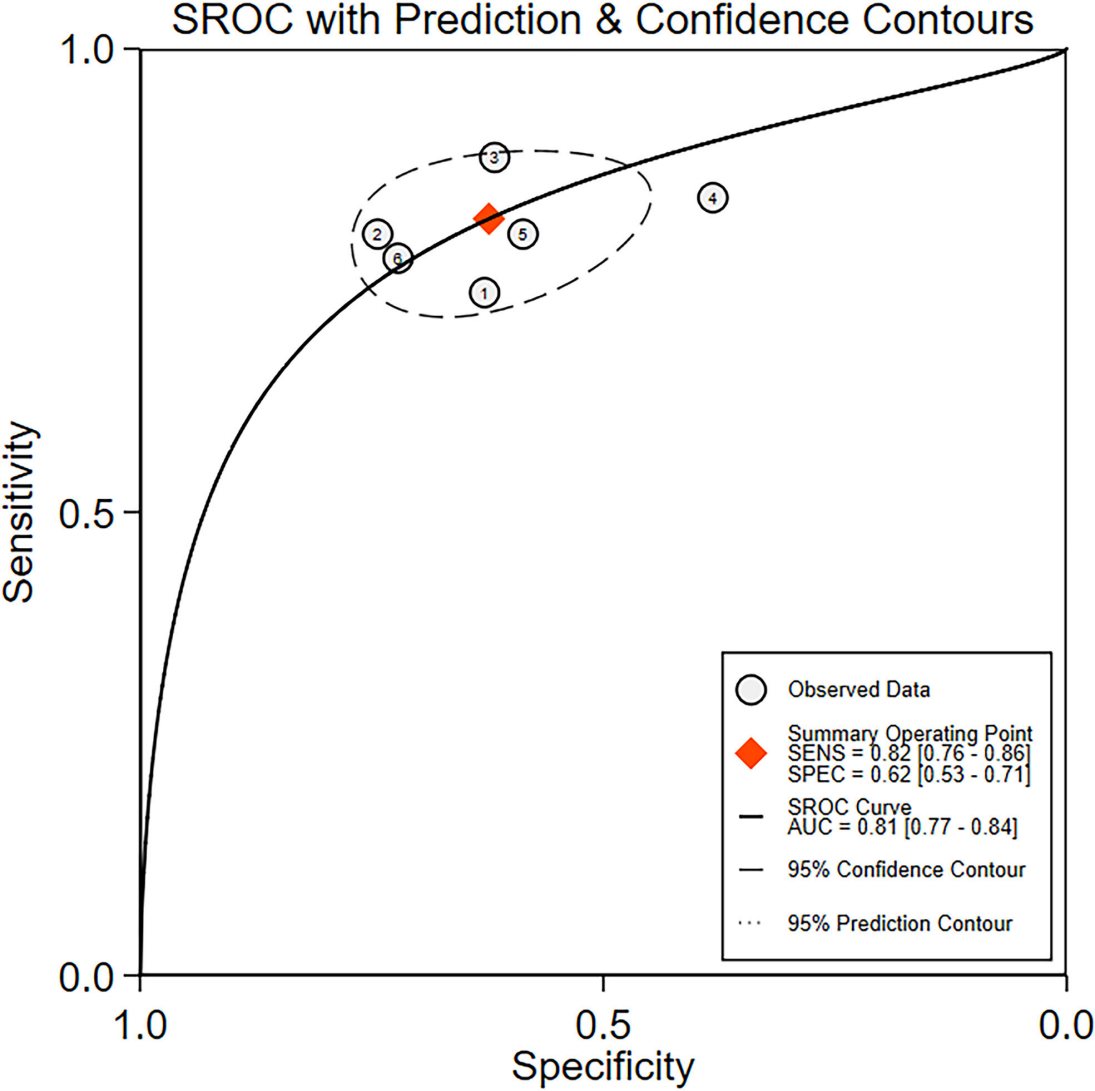
The summary receiver operating characteristic (SROC) curve based on circRNAs for diagnosis analysis. ROC, receiver operator characteristic.

**Figure 5:**
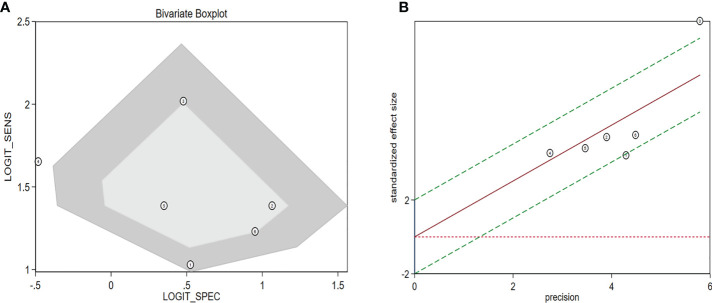
Assessment of the diagnostic accuracy of circRNAs in PCa. **(A)** Bivariate boxplot. **(B)** Galbraith plot. circRNAs, circular RNAs; PCa, prostate cancer.

**Figure 6:**
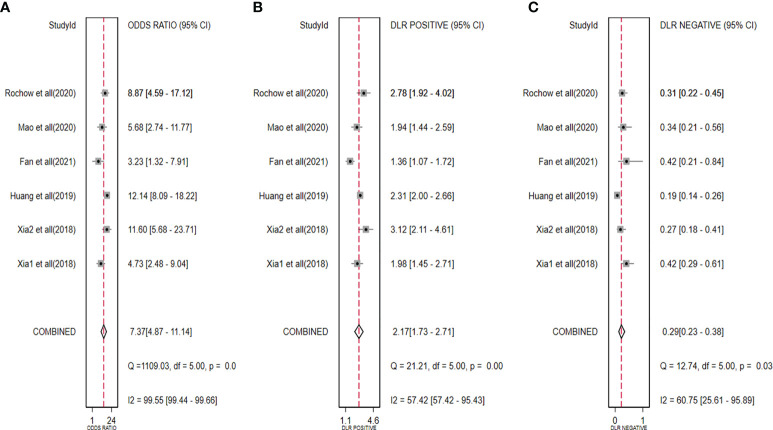
Forest plots for pooled DOR **(A)**, PLR **(B)**, and NLR **(C)** of circRNAs for PCa. DOR, diagnostic odds radio; PLR, positive likelihood ratio; NLR, negative likelihood ratio; circRNAs, circular RNAs; PCa, prostate cancer.

The diagnostic meta-analysis included 1,024 patients from five studies that were all qualified. The pooled SEN and SPC were calculated to examine the diagnostic utility of circRNAs, and the findings are shown in **Figure 3**. A random-effects model was used because of the observable heterogeneity (I^2^ = 67.42% and I^2^ = 77.73%). As the results showed, the pooled SPC was 0.82 (95% CI: 0.76–0.86), whereas the pooled SEN was 0.62 (95% CI: 0.53–0.71). Moreover, the AUC was 0.81 (95% CI: 0.77–0.84) according to the SROC curve analysis (**Figure 4**). Thus, it could be seen that these circRNAs had excellent diagnostic efficacy in distinguishing patients with PCa from the healthy population. As shown in the bivariate boxplot and Galbraith plot (**Figures 5A, B**), one study fell outside the game and Galbraith diagrams, respectively, which suggested that there was heterogeneity in the analysis. Because of the limited inclusion of articles, a subgroup analysis could not be performed to find the sources of heterogeneity. In addition, the DOR was 7.37 (95% CI: 4.87–11.14). The pooled PLR and NLR were 2.17 (95% CI: 1.73–2.71) and 0.29 (95% CI: 0.23–0.38), respectively (**Figures 6A–C**). These results also demonstrated the excellent diagnostic ability of circRNAs in patients with PCa. These findings suggested that circRNAs have good diagnostic accuracy for PCa when taken combined.

### Clinicopathological parameters

The association between circRNAs and clinicopathological features is shown in **Table 3**. For clinicopathological features, 16 studies were enrolled in our meta-analysis with a total of 1,153 patients. Upregulation of carcinogenic circRNAs was linked to adverse clinical characteristics (Gleason score: OR = 0.222, 95% CI: 0.145–0.340; T classification: OR = 0.274, 95% CI: 0.175–0.430; lymph node metastasis: OR = 0.353, 95% CI: 0.175–0.716; tumor size: OR = 0.226, 95% CI: 0.099–0518). Furthermore, decreased expression of tumor-suppressor circRNAs was also linked to worse clinical outcomes (Gleason score: OR = 1.689, 95% CI: 1.144–2.493; T classification: OR = 2.586, 95% CI: 1.779–3.762);. Remarkably, there was no correlation between circRNA expression and other clinicopathologic factors such as age, TNM stage, distant metastasis, expression of PSA, and smoking.

### Prognosis analysis

**Figure 7:**
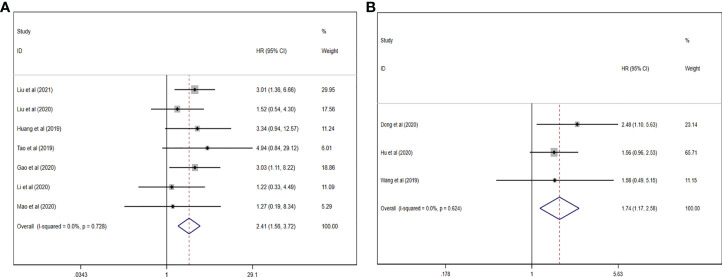
Forest plots for the association between circRNAs and estimating HR in PCa. **(A)** Upregulated circRNAs. **(B)** Downregulated circRNAs. HR, hazard ratios; PCa, prostate cancer; circRNAs, circular RNAs.

In our work, the prognosis meta-analysis included 1,164 participants from 10 investigations, all of which were eligible. The role of upregulated circRNAs in PCa prognosis was estimated using fixed-effect models (I^2^ = 0.0%, p = 0.782), and the results demonstrated that upregulation of carcinogenic circRNAs was linked to a poor prognosis (HR = 2.408, 95% CI: 1.559–3.720, p < 0.001) (**Figure 7A**). At the same time, downregulation of tumor-suppressor circRNAs was significantly associated with worse PCa prognosis (HR = 1.739, 95% CI: 1.147–2.576, p = 0.006). Because there was no heterogeneity between studies (I^2^ = 0%, p = 0.624), a fixed-effects model was used (**Figure 7B**).

### Publication bias and sensitivity analysis

**Figure 8:**
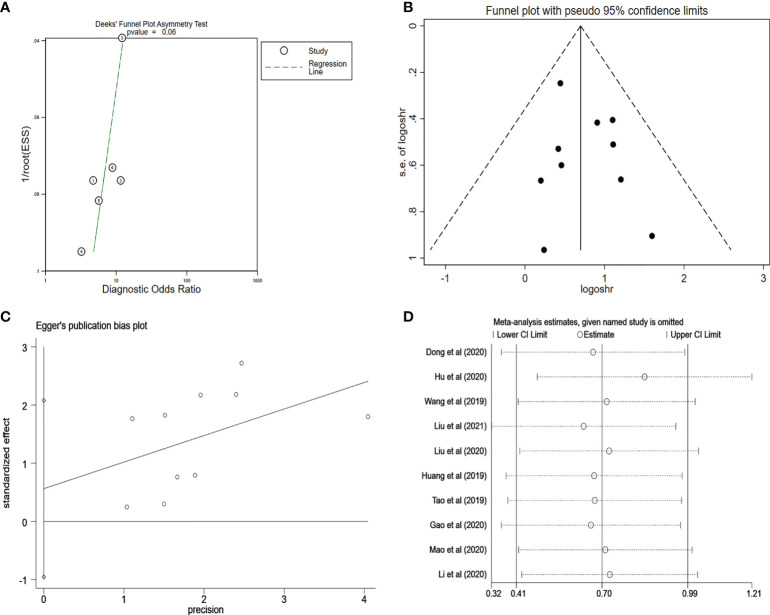
**(A)** Deeks’ funnel plot for evaluating the publication bias of the included study on the diagnosis analysis. **(B)** Funnel plot for evaluating the publication bias of the included study on the prognosis analysis. **(C)** Egger’s funnel plot for evaluating the publication bias of the included study on the prognosis analysis. **(D)** Sensitivity analysis for evaluating the influence of the omitted study on the pooled HR. PCa, prostate cancer; HR, hazard ratio.

We used the Deeks’ funnel plot asymmetry test to analyze the potential publication bias of diagnostic meta-analysis and found that there was no clear publication bias (p = 0.07) (**Figure 8A**). Furthermore, the potential publication bias of prognostic meta-analysis was investigated, which showed no publication bias in the included studies (**Figure 8B**). In addition, the Egger’s test also supported the conclusion that there was no publication bias (p = 0:85, **Figure 8C**). At the same time, in our research, sensitivity analysis revealed that the pooled results in the prognostic meta-analysis were stable (**Figure 8D**).

## Discussion

In recent years, the high incidence and mortality of PCa have caused worldwide concern (41, 42). The diagnosis and prognosis of PCa are currently determined by tissue biopsy and PSA levels (42). However, tissue biopsy is invasive, and the predicted accuracy of PSA should be furthermore improved. Therefore, PSA has been steadily discouraged from being advised in recent years (43, 44).

Many researchers are looking for new biomarkers to determine the diagnosis and prognosis of patients with PCa to enhance their survival (45). circRNAs are non-coding RNAs playing an important role in the development and progression of cancer (46). At the same time, circRNAs were thought to have promising prospects and advantages as perfect biomarkers for human cancer diagnosis and prognosis (47).

Therefore, as a novel biomarker, circRNAs have several advantages for clinical applications. First, traditional puncture biopsy entails a risk of injuries and is more difficult for the operator to perform. In contrast, circRNAs in plasma are more readily available and harmless. Second, circRNAs are resistant to denaturation because of their stable structures and conservative sequences. Third, circRNAs outperform standard markers in terms of diagnostic and prognostic value, as well as accuracy.

The aberrant expression of circRNAs has been shown in numerous investigations in patients with PCa; with a few studies, few meta-analyses have been published on the role of circRNAs in PCa diagnosis or prognosis. Our study is the first to discuss the link between circRNA expression and diagnostic performance, prognostic value, and clinical characteristics in PCa.

In our meta-analysis, the aggregated data revealed an AUC of 0.81, with a sensitivity of 0.82 and a specificity of 0.62 for diagnostic value, indicating that circRNAs could be employed as diagnostic biomarkers for PCa. In terms of clinical and prognostic importance, abnormal expression of circRNAs was connected to clinical parameters and prognosis.

The sensitivity and specificity results also demonstrated that circRNAs could discriminate between healthy people and patients, but the specificity needs to be enhanced. However, the bivariate boxplot and Galbraith plot results indicated that the studies included in the diagnostic analysis were heterogeneous. Because the number of included papers was insufficient to allow additional subgroup analysis to identify sources of heterogeneity, further research is warranted.

In clinical practice, the PLR and NLR signify diagnostic ability. The PLR > 10 and NLR < 0.1 are considered to indicate good diagnostic ability (47). However, in our study, PLR was 2.17 and NLR was 0.29, which meant that circRNAs’ diagnostic accuracy is currently limited. In other words, circRNAs would give a higher rate of FN and FP in clinical applications.

DOR is a measurement index for diagnostic performance that incorporates the advantages of sensitivity and specificity. The higher the value of DOR, the better it can identify test performance (48). In our study, the pooled DOR was 7.37, which supported the use of circRNAs as a viable diagnostic tool for PCa.

As for clinicopathological parameters, T classification and Gleason score were found to be linked with both upregulated and downregulated circRNAs. The aberrant expression of circRNAs in colorectal carcinoma and esophageal cancer was also linked to T classification in the studies by Yuan et al. (49) and Lin et al. (50). Together, this could imply that circRNAs are vital for tumor staging.

The Gleason score is a widely used method for histological grading of PCa and a useful tool for making plans for PCa treatment (51). Our findings revealed that circRNAs were highly linked to a low Gleason score, indirectly reflecting the status of PCa.

In terms of prognostic values, our recent meta-analysis found that aberrant circRNA expression was strongly linked to poor overall survival. As a result, prompt monitoring of circRNA alterations in patients with PCa can aid clinical decision-making and help patients live longer.

Some studies focusing on experimental research rather than cohort or case-control research also demonstrated that circRNA does play a role in the diagnosis and prognosis of PCa. As described by Vo et al., multiple upregulated or downregulated circRNAs were shown to be associated with PCa progression (52). Their results are consistent with previous studies showing that circRNAs generally do not directly contribute to the growth of cancer cells but instead do so indirectly through controlling mRNAs (53–55). These studies support the value of circRNA in PCa; however, we did not include them in our meta-analysis because most of them are clustered in experiments that lack data from entire cohort studies.

Numerous studies about the effect of circRNAs on various cancers have been conducted. Some meta-analyses had demonstrated that circRNA had good diagnostic and prognostic value for patients with colorectal carcinoma (49), solid cancers (56), and osteosarcoma (57). However, only several meta-analyses exploring the diagnostic and prognostic value of circRNA in PCa have been conducted to date. Yuan et al. performed a meta-analysis that included only articles from 2015 to 2019 (49). However, in the last 3 years, more studies have focused on the differences in circRNA expression between patients with cancer patients and healthy subjects. Our study fills this gap and covers a wider range of data. In addition, Li et al. included only five studies in their prognosis meta-analysis (56). This meta-analysis was based on a small number of studies, which may have introduced some bias. The present study has been quantitatively supplemented to make the results of the meta-analysis more convincing. In addition, the source of specimens was indicated in our meta-analysis. Compared with previous meta-analyses, we excluded the effect of heterogeneity due to specimen source. Thus, our study is an update and addition to similar meta-analyses evaluating the role of circRNA in PCa and provides a grounding for related research.

There are still limitations in our current meta-analysis. First, we only included five papers in our diagnostic meta-analysis, and the small number of research has limited the popularization of circRNAs in clinical applications. Second, because of the restricted amount of data from included studies, we were unable to run a subgroup analysis to analyze the source of heterogeneity in the diagnostic meta-analysis. Furthermore, some research did not provide clear HR data. We retrieved necessary data from provided KM curves, which might have caused some bias. Finally, because the majority of the research examined was from China, the external application of our findings across diverse areas may be hampered. To ensure the accuracy and relevance of the findings, a comprehensive research is required.

## Conclusion

Together, our meta-analysis revealed that circRNAs have moderately high diagnostic accuracy for PCa. The results of the prognostic meta-analysis revealed that aberrant expression of circRNAs is significantly associated with poor prognostic outcomes and clinicopathological values. Therefore, circRNAs can be useful indicators for the diagnosis and prognosis of PCa. However, further studies with more multicenter data, as well as high-quality studies, are needed to demonstrate the role of circRNAs in PCa.

## Data availability statement

The original contributions presented in the study are included in the article/**Supplementary Material**. Further inquiries can be directed to the corresponding author.

## Author contributions

JX and HJ contributed to conceive this study, performed quality assessment of included studies, analyze the data, and write the manuscript. YZ analyzed the data and performed quality assessment of included studies. LZ and ZZ reviewed the manuscript. JL provided the financial support. All authors contributed to manuscript revision, read, and approved the submitted version.

## Funding

This manuscript was supported by the grants from Sichuan Science and Technology Program (2021YFS0332), and Southwest Medical University Science Program (00031839).

## Conflict of interest

The authors declare that the research was conducted in the absence of any commercial or financial relationships that could be construed as a potential conflict of interest.

## Publisher’s note

All claims expressed in this article are solely those of the authors and do not necessarily represent those of their affiliated organizations, or those of the publisher, the editors and the reviewers. Any product that may be evaluated in this article, or claim that may be made by its manufacturer, is not guaranteed or endorsed by the publisher.
