# Supplementary material for: Prognostic and diagnostic value of circRNA expression in prostate cancer: A systematic review and meta-analysis
Source: Front Oncol. 2022 Nov 7;12:945143. doi: 10.3389/fonc.2022.945143 (PMC9676972; doi:10.3389/fonc.2022.945143)
Supplement: Supplementary file 1 [file DataSheet_1.pdf]

Prognostic and diagnostic value of circRNA expression in prostate cancer: a systematic review and meta-analysis

Jingling Xie[1†], Hui Jiang[1†], Yuanqing Zhao[1], Xin rui Jin[1], Baolin Li[1], Zixin Zhu[1], Limei Zhang[1], Jinbo Liu[1*]

[1] Department of Laboratory Medicine, the Affiliated Hospital of Southwest Medical University, 25 Taiping Street, Luzhou, 646000, Sichuan, P.R. China.

[†]These authors have contributed equally to this work and share first authorship.

Tel: +86 0830 3165730

Fax: +86 0830 3165730

*** Correspondence:**

Jinbo Liu

liujb7203@swmu.edu.cn

Detailed search strategy:

Web of science:

TS=(Prostatic Neoplasms OR Prostate Neoplasms OR Neoplasms, Prostate OR Neoplasm, Prostate OR Prostate Neoplasm OR Neoplasms, Prostatic OR Neoplasm, Prostatic OR Prostatic Neoplasm OR Prostate Cancer OR Cancer, Prostate OR Cancers, Prostate OR Prostate Cancers OR Cancer of the Prostate OR Prostatic Cancer OR Cancer, Prostatic OR Cancers, Prostatic OR Prostatic Cancers OR Cancer of Prostate AND RNA, Circular OR circRNAs OR Closed Circular RNA OR Circular RNA, Closed OR RNA, Closed Circular OR Circular RNA OR Circular RNAs OR RNAs, Circular OR circRNA OR Circular Intronic RNA OR Intronic RNA, Circular OR RNA, Circular Intronic OR CiRNA)
TS= (RNA, Circular OR circRNAs OR Closed Circular RNA OR Circular RNA, Closed OR RNA, Closed Circular OR Circular RNA OR Circular RNAs OR RNAs, Circular OR circRNA OR Circular Intronic RNA OR Intronic RNA, Circular OR RNA, Circular Intronic OR CiRNA)

Pubmed:

((((((((((((RNA, Circular[MeSH Terms]) OR (circRNAs[Title/Abstract])) OR (Closed Circular RNA[Title/Abstract])) OR (Circular RNA, Closed[Title/Abstract])) OR (RNA, Closed Circular[Title/Abstract])) OR (Circular RNA[Title/Abstract])) OR (Circular RNAs[Title/Abstract])) OR (RNAs, Circular[Title/Abstract])) OR (circRNA[Title/Abstract])) OR (Circular Intronic RNA[Title/Abstract])) OR (Intronic RNA, Circular[Title/Abstract])) OR (RNA, Circular Intronic[Title/Abstract])) OR (CiRNA[Title/Abstract])) AND (((((((((((((((((Prostatic Neoplasms[MeSH Terms]) OR (Prostate Neoplasms[Title/Abstract])) OR (Neoplasms, Prostate[Title/Abstract])) OR (Neoplasm, Prostate[Title/Abstract])) OR (Prostate Neoplasm[Title/Abstract])) OR (Neoplasms, Prostatic[Title/Abstract])) OR (Neoplasm, Prostatic[Title/Abstract])) OR (Prostatic Neoplasm[Title/Abstract])) OR (Prostate Cancer[Title/Abstract])) OR (Cancer, Prostate[Title/Abstract])) OR (Cancers, Prostate[Title/Abstract])) OR (Prostate Cancers[Title/Abstract])) OR (Cancer of the Prostate[Title/Abstract])) OR (Prostatic Cancer[Title/Abstract])) OR (Cancer, Prostatic[Title/Abstract])) OR (Cancers, Prostatic[Title/Abstract])) OR (Prostatic Cancers[Title/Abstract])) OR (Cancer of Prostate[Title/Abstract]))

EMBASE:

No.	Query	Result
#19	#14 AND #18	170
#18	#15 OR #16 OR #17	8154
#17	'rna, circular':ab,ti	76
#16	'circular rna':ab,ti	4151
#15	'circular ribonucleic acid'/exp	6781
#14	#1 OR #2 OR #3 OR #4 OR #5 OR #6 OR #7 OR #8 OR #9 OR #10 OR #11 OR #12 OR #13	244917
#13	'prostatic malignancy':ab,ti	229
#12	'prostatic cancer':ab,ti	8119
#11	'prostate malignant tumour':ab,ti	2
#10	'prostate malignant tumor':ab,ti	5
#9	'prostate malignant neoplasm':ab,ti	2
#8	'prostate malignancy':ab,ti	299
#7	'prostate gland cancer':ab,ti	23
#6	'malignant prostatic tumour':ab,ti	3
#5	'malignant prostatic tumor':ab,ti	4
#4	'malignant prostate tumour':ab,ti	3
#3	'malignant prostate tumor':ab,ti	14
#2	'cancer, prostate':ab,ti	2254
#1	'prostate cancer'/exp	241557

PICOS Principles:

1. **Diagnostic:**

P(Populations): Prostate cancer patients
I (Interventions): Levels of circRNA
C (Comparators): Health Volunteers
O (Outcomes): Diagnosed with prostate cancer
S (Study designs): Cohort or case-control research

2. **Clinicopathological features:**

P(Populations): Prostate cancer patients
I (Interventions): Levels of circRNA
C (Comparators): Health Volunteers
O (Outcomes): Clinicopathological features of tumors
S (Study designs): Cohort or case-control research

3. **Prognosis:**

P(Populations): Prostate cancer patients
I (Interventions): Levels of circRNA
C (Comparators): Health Volunteers
O (Outcomes): Survival rates
S (Study designs): Cohort or case-control research
